# Validation of ELISA with recombinant antigens in serological diagnosis of canine *Leishmania infantum* infection

**DOI:** 10.1590/0074-02760200428

**Published:** 2021-03-12

**Authors:** Mahyumi Fujimori, Arleana do Bom Parto Ferreira de Almeida, Stella Maria Barrouin-Melo, Luiz Ricardo Paes de Barros Cortez, Malcolm Scott Duthie, Roberto Mitsuyoshi Hiramoto, Flaviane Alves de Pinho, Steven Gregory Reed, Valéria Régia Franco Sousa, Nazaré Fonseca Souza, Rodrigo Martins Soares, José Eduardo Tolezano, Maria Carmen Arroyo Sanchez, Hiro Goto

**Affiliations:** 1 Instituto de Medicina Tropical da Faculdade de Medicina da Universidade de São Paulo, Laboratório de Soroepidemiologia e Imunobiologia, São Paulo, SP, Brasil; 2 Universidade Federal de Mato Grosso, Faculdade de Medicina Veterinária, Departamento de Clínica Médica Veterinária, Cuiabá, MT, Brasil; 3 Universidade Federal da Bahia, Hospital-Escola de Medicina Veterinária, Laboratório de Infectologia Veterinária, Salvador, BA, Brasil; 4 Secretaria Municipal de Saúde de Bauru, Departamento de Saúde Coletiva - Vigilância Ambiental, Bauru, SP, Brasil; 5 Host Directed Therapeutics, Seattle, WA, United States of America; 6 Instituto Adolfo Lutz, Centro de Parasitologia e Micologia, Núcleo de Parasitoses Sistêmicas, São Paulo, SP, Brasil; 7 Universidade Federal Rural da Amazônia, Instituto da Saúde e Produção Animal, Belém, Pará, Brasil; 8 Universidade de São Paulo, Faculdade de Medicina Veterinária e Zootecnia, Departamento de Medicina Veterinária Preventiva e Saúde Animal, Laboratório de Doenças Parasitárias, São Paulo, SP, Brasil; 9 Universidade de São Paulo, Faculdade de Medicina, Departamento de Medicina Preventiva, São Paulo, SP, Brasil

**Keywords:** canine visceral leishmaniasis, diagnosis, recombinant antigens, ELISA, epidemiological inquiry

## Abstract

**BACKGROUND:**

Dogs are the main peridomiciliary reservoir of *Leishmania infantum* thus the correct diagnosis of infection is essential for the control of the transmission and treatment as well. However, the diagnosis is based on serological assays that are not fully effective.

**OBJECTIVE:**

We aimed to establish an effective serological assay for the diagnosis of *L. infantum* infected dogs using *Leishmania*-derived recombinant antigens.

**METHODS:**

*Leishmania* derived rK39-, rK28-, rKR95-based enzyme-linked immunosorbent assay (ELISA) was standardized using symptomatic and asymptomatic *L. infantum*-infected dogs. Then 2,530 samples from inquiry in endemic areas for VL were evaluated and the results compared with recommended assays by the Brazilian Ministry of Health (MH algorithm). Further samples from a cohort of 30 dogs were searched.

**FINDINGS:**

For rK39-, rK28- and rKR95-ELISA the sensitivity was around 97% and specificity 100%. The positivity of these three ELISA in the inquiry samples was 27-28%, around 10% higher than the assays currently in use. When cohort samples were searched, we observed likely false-negative results (> 65%) with supposedly negative samples that turned positive six months later with the assays in use (MH algorithm).

**MAIN CONCLUSIONS:**

For the diagnosis of *L. infantum*-infected dogs, rK39-based ELISA showed better diagnostic performance than other assays in use in Brazil and worldwide.

Leishmaniases are a family of infectious diseases caused by obligatory intracellular protozoa of the genus *Leishmania*. In 2017, 94% of new leishmaniasis cases reported by the World Health Organization (WHO) were concentrated in seven countries: Brazil, Ethiopia, India, Kenya, Somalia, South Sudan and Sudan.^1^ Visceral leishmaniasis (VL) is the most severe form of the disease that, if left untreated, leads to the death of more than 95% of individuals.^2^ VL is caused by *Leishmania donovani* in India, East Africa, Bangladesh and Nepal in an anthroponotic cycle, and *L. infantum* in China, Central Asia, the Mediterranean, part of Africa and Latin America in a zoonotic cycle.^3^ In the Americas, VL occurs in more than 12 countries, but 96% of the cases reported between 2001 and 2017 occurred in Brazil.^4^

Dogs (*Canis familiaris*) are the main *L. infantum* reservoir in urban and peri-urban areas in several regions of the world^5^ and their infection is known to precede the occurrence of human disease.^6^ Canine leishmaniasis (CanL) is one of the most important vector-borne diseases, and over 70 countries have the endemic canine disease.^7^ Thus, the early diagnosis of infected animals is essential for the treatment or monitoring of VL transmission by surveillance programs.

*Leishmania* infected dogs typically present high anti-*Leishmania* IgG antibody titers, and serological methods have been widely used as an indirect detection method.^5^ In Brazil, the Ministry of Health incorporated a diagnostic algorithm that uses the dual-path platform (DPP) immunochromatographic test for *Leishmania* for primary screening and enzyme-linked immunosorbent assay (ELISA) detecting antibodies against the total *L. major*-like antigen, for confirming the diagnosis of CanL.^8^ ^,^ ^9^ ^,^ ^10^ ^,^ ^11^ ^,^ ^12^ The necessity to confirm the diagnosis after screening by the DPP test was suggested in the study of Figueiredo et al.,^10^ showing a decrease in sensitivity (75%) and low specificity (72%) in asymptomatic dogs.

On the total *L. major*-like-ELISA, since it is prepared from the crude lysate of *L. major*-like parasites, variability is frequent in performance, with reported sensitivities ranging from 11.59%^13^ to 100.0%^14^ and specificities ranging from 68.0%^14^ to 90.74%^13^ In this scenario, although there is a faster diagnosis of CanL using the immunochromatographic test as screening, we see the possibility of improving the ELISA that currently employs *L. major*-like total lysate. It would be desirable to have an ELISA based on a recombinant antigen as it is a more specific protein, and production can be done on an industrial scale. Thus, we propose in this study to evaluate the use of four recombinant proteins (rK39, rK28, rKR95 and rK18) for the serological diagnosis of dogs in ELISA.

The recombinant protein rK39 is a repeated sequence of 39 amino acids highly preserved in *L. infantum* and *L. donovani*, related to kinesin and highly expressed in amastigotes.^15^ In humans, rK39-ELISA detects active VL and asymptomatic *Leishmania* infection^16^ with sensitivity in the range of 69.4-97.6% and specificity of 81.0-100.0%, depending on geographical area.^17^ ^,^ ^18^ ^,^ ^19^ In dogs, rK39-ELISA has yielded 95.0% sensitivity and 100.0% specificity.^20^ In a multicentric study carried out in Italy,^21^ rK39-ELISA presented 97.1% sensitivity and 99.4% specificity. In the latter study, the serological inquiry’s performance was not shown clearly and was compared with the indirect immunofluorescent (IFI) anti-*Leishmania* antibody assay that we know has poor performance.

The rK28 protein, constructed as a chimeric protein based on the fusion of *L. infantum* rK9, rK26 and rK39 epitopes, presented high sensitivity (99%) and specificity (96%) in the ELISA in samples of dogs from Italy.^22^ In human VL, the rK28-ELISA showed a sensitivity of 96.8-99.6% and specificity of 96.2-100.0%.^23^ ^,^ ^24^ In Latin America, immunoenzymatic tests developed with rK28 for the CanL diagnosis showed a sensitivity of 91.0% in the qualitative and quantitative ELISA and specificity of 100% in qualitative and 98.7% in the quantitative assay.^25^

Presently other promising recombinant *Leishmania* antigens that we include in this project became available, and it would be important to study them in other geographical regions, including Brazil, since regional variation in infection features may present.

The recombinant antigen KR95 is a kinesin-bound protein comprising peptide sequences identified from *L. donovani* infected patients with no corresponding homology in humans. Besides, although the identity between KR95 and *Trypanosoma cruzi* kinesin is 79%, there appears to be no cross-reactivity.^26^ Finally, rK18 was established as useful for both diagnosis and treatment follow up, with a decrease in antibody concentrations over 180 days after treatment.^27^

Parasitological examinations that require invasive procedures were not performed on canine survey samples. Instead, the sera were subjected to the *Leishmania* direct agglutination test (DAT) which has good diagnostic performance, both in human and canine VL. In a study with patients from Ethiopia and Brazil, DAT had 100% sensitivity and specificity.^28^ In CanL in the Mediterranean region, DAT reached 100% sensitivity and 98.9% specificity,^29^ while in Venezuela the DAT sensitivity was 92.59% (75.69-99.09%), specificity was 100% (79.4-100.0%).^30^ Thus, the DAT was our reference test for comparative purposes with the established protocol by the Ministry of Health and also with ELISAs tests with recombinant antigens.

In this study, we validated the ELISA with recombinant *Leishmania* antigens, suggesting the rK39 antigen for the diagnosis of CanL combined with immunochromatographic DPP assay or alone. Although the rK39-ELISA test has been evaluated in European dogs,^21^ ^,^ ^31^ demonstrating good sensitivity and specificity, there are no studies on the standardization and validation of the ELISA with a large panel of dog samples in Brazil. Therefore, the knowledge of these data is made necessary to establish evidence on the performance of the CanL serological diagnosis in the studied regions. Further, the rK39-ELISA here shown with better diagnostic performance can be extended for diagnosis and management of CanL worldwide.

## MATERIALS AND METHODS

*Samples* - Samples from various institutions in different regions of Brazil were used. Samples were provided by Instituto Adolfo Lutz de São Paulo (IAL/SP), Faculdade de Agronomia, Medicina Veterinária e Zootecnia da Universidade Federal de Mato Grosso (UFMT), Instituto da Saúde e Produção Animal da Universidade Federal Rural da Amazônia (UFRA), Escola de Medicina Veterinária e Zootecnia da Universidade Federal da Bahia (UFBA) and Faculdade de Medicina Veterinária e Zootecnia da Universidade de São Paulo (USP). To calculate the sensitivity, specificity and cut-off of the ELISA with recombinant antigens and to determine the cut-off of the DAT, the following samples were obtained from the IAL/SP, where the parasitological examination, DPP and *L. major*-like-ELISA (characterized samples) were performed:

- Sera from 74 symptomatic dogs from areas with the canine transmission of *L. infantum* and the local presence of the vector for *Leishmania* (Fernandópolis - SP, Votuporanga - SP and Santa Fé do Sul - SP) attended at the Zoonosis Control Centers were used. These dogs had a positive parasitological exam in sternum bone marrow and popliteal lymph node aspirates on glass slides that were fixed and stained with a modified Romanowsky staining rapid test kit (Panótico Rápido, Laborclin, Brazil). In our study, animals with a positive parasitological (gold standard) result were included regardless of the serological result.

- Sera from 66 healthy dogs from areas without canine transmission of *L. infantum*, with the absence of the vector for *Leishmania* (Jundiaí - SP). These dogs were negative by the tests recommended in the algorithm of the Brazilian Ministry of Health (DPP and ELISA-*L. major*-like) and DAT.

To evaluate whether subclinically VL infected dogs could be detected by recombinant antigen-ELISA, sera from 19 asymptomatic dogs were obtained from areas with the canine transmission of *L. infantum* and the local presence of the *Leishmania* vector (Fernandópolis - SP, Votuporanga - SP and Santa Fé do Sul - SP). These dogs were positive by the tests recommended in the algorithm of the Brazilian Ministry of Health (DPP and ELISA-*L. major*-like) and DAT.

**Fig. 1:**
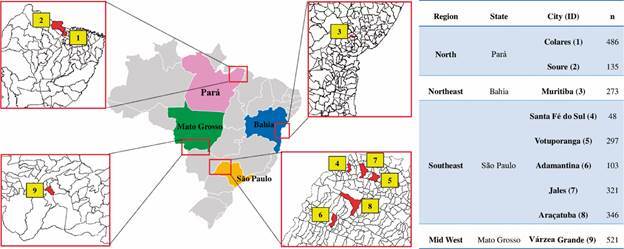
serological inquiry samples of endemic areas for canine leishmaniasis in Brazil. Samples were collected from 2,530 dogs from municipalities of the various geographical regions. ID: city identification; n: number of samples.

For the validation of each ELISA, samples collected in serological inquiry were used to search for *L. infantum* infection in municipalities from north, northeast, southeast and mid-west of Brazil, endemic for CanL. Samples were collected from 2,530 dogs from municipalities of the State of Pará (Colares, city 1: n = 486 and Soure, city 2: n = 135), State of Bahia (Muritiba, city 3: n = 273), State of São Paulo (Santa Fé do Sul, city 4: n = 48, Votuporanga, city 5: n = 297, Adamantina city 6: n = 103, Jales, city 7: n = 321 and Araçatuba, city 8: n = 346) and State of Mato Grosso (Várzea Grande, city 9: n = 521) (Fig. 1).

To evaluate if an infection could be detected earlier than the recommended assays currently in use, paired-samples from 30 dogs in the municipality of Bauru (located in the State of São Paulo) were obtained at day 0 (time 1) and again at 180 days (time 2) for evaluation. These dogs used collars impregnated with deltamethrin (Scalibor - Intervet) to reduce the risk of a new infection during the period of evaluation. The collars were changed according to the manufacturer’s recommendation or replaced in case of losses, in order to avoid infection through contact with the vector.

To evaluate if the recombinant antigens cross-react with other diseases, we selected 10 samples from dogs without clinical signs for CanL but reagents for *Ehrlichia canis* and non-reagents for *Borrelia burgdorferi*, *Anaplasma phagocytophilum*, *Anaplasma platys* and *Dirofilaria immitis* in SNAP 4DX Plus^®^ test (IDEXX Laboratories Inc., Westbrook, ME, USA). These animals came from São Paulo, a non-endemic municipality for VL.

*Sample size calculation* - Aiming at a 95% sensitivity for the rK39-ELISA, rK28-, rKR95- and rK18- tests^32^ and considering a 95% confidence interval (95% CI) of ± 5%, the sample size needed was at least 73 VL dogs. Aiming at a 98% specificity, at least healthy 47 controls were required,^32^ considering a 95% CI of ± 4%.^33^ For the ELISA’s validation with recombinant antigens, the sample size was calculated according to Banoo et al.^33^ Considering a 95% confidence level, an infection prevalence of 15% and an error of 5%, the sample size defined for the study of each region was 196 dogs.

*Ethical aspects of the research* - The project was approved by the Research Ethics Committee on the Use of Animals of the Instituto de Medicina Tropical, Universidade de São Paulo (CEUA - IMT/USP) on 02/06/2015 (Process number 000305A). The necessary care was taken during the collection of samples, preserving the anonymity of the owners and dogs and regarding conservation and correct use of the stored biological material.

Serological tests

**Fig. 2:**
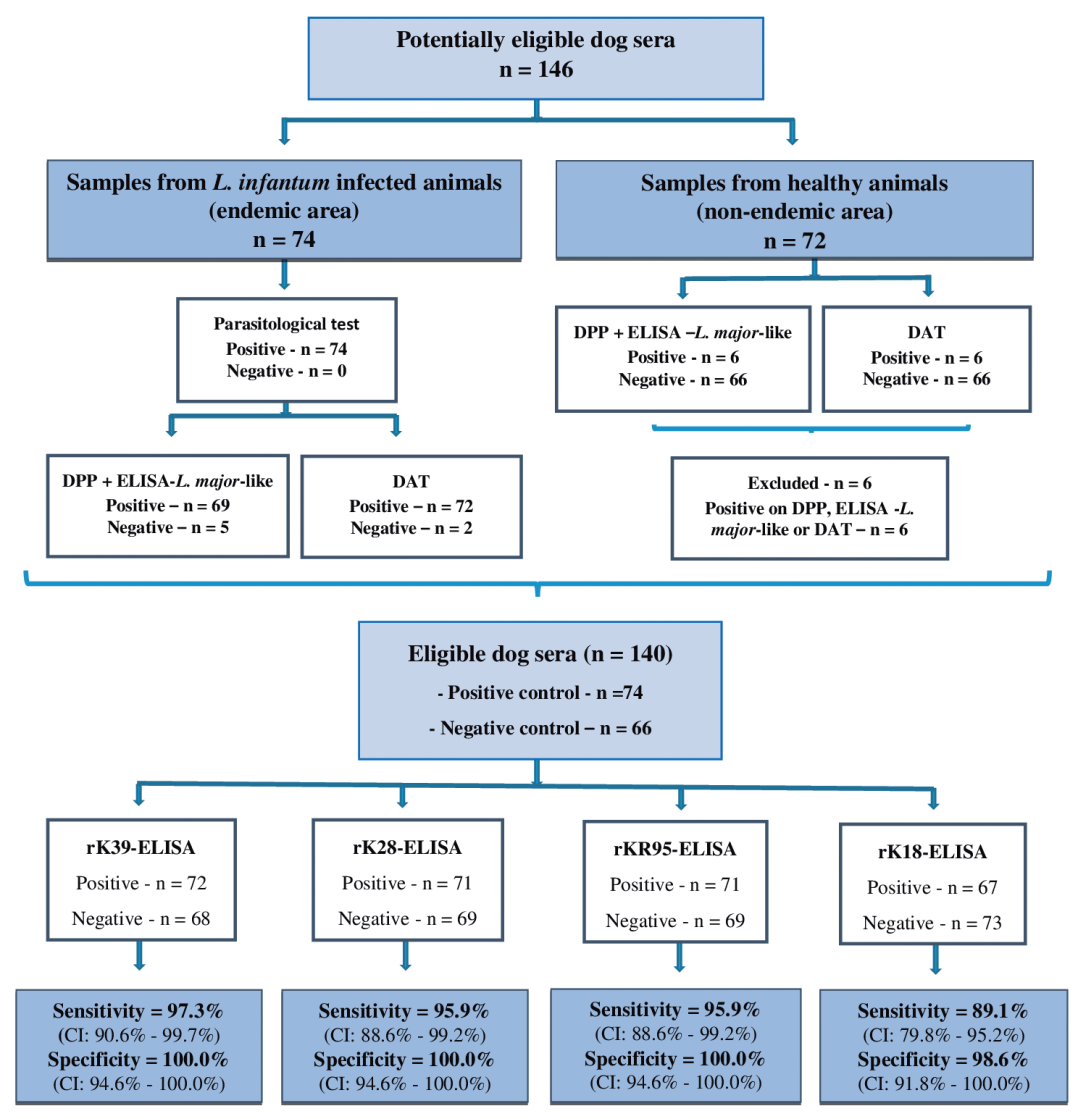
flowchart showing the composition of the positive and negative controls. For the positive controls, the parasitological, dual-path platform (DPP) and enzyme-linked immunosorbent assay (ELISA)-*Leishmania major*-like tests were performed. For negative controls, DPP and *L. major*-like-ELISA tests were performed. Positive and negative control samples were subjected to the rK39-ELISA, rK28-ELISA, rKR95-ELISA and rK18-ELISA. The results were used to construct the receiver operating characteristic (ROC) curves and to obtain the specificity and sensitivity values. n: number of samples; DAT: direct agglutination test.

- DPP - TR DPP^®^ Leishmaniose Visceral Canina (Bio-Manguinhos, Fiocruz, Rio de Janeiro, Brazil) - The immunochromatographic tests used by the *L. infantum* Infection Control Program, of the São Paulo Department of Health, and the Ministry of Health from Brazil to screen dogs for infections was used according to the manufacturer’s recommendations.

- *L. major*-like-Elisa - EIE Leishmaniose Visceral Canina (Bio-Manguinhos, Fiocruz, Rio de Janeiro, Brazil) - The ELISA, used by the *L. infantum* Infection Control Program, of the São Paulo Department of Health, and the Ministry of Health from Brazil to confirm the positive DPP^®^ test, was performed according to the manufacturer’s recommendations. The Ministry of Health recommends that only samples positive by DPP should be tested by *L. major*-like-ELISA for confirmation. However, in this study, all samples were submitted to this ELISA.

- DAT (Institute of Tropical Medicine - Antwerp/Belgic) - DAT was performed at the Instituto de Medicina Tropical, Universidade de São Paulo, according to the manufacturer’s recommendations. To calculate the agglutination test cut-off, the same groups (Fig. 2), as reported above, were used. Control samples were diluted in series (factor 2) ranging from 1/20 through 1/20,480, and from the positive and negative sample titers, the receiver operating characteristic (ROC) curve was constructed, obtaining the DAT sensitivity, specificity, and cut-off values. In the canine survey of the evaluated regions, the canine sera were also diluted in series (factor 2) ranging from 1/20 through 1/20,480.

- ELISA with recombinant antigens- rK39, rK28, rKR95 and rK18 - The recombinant antigens rK39, rK28, KR95 and rK18 were produced at the Infectious Disease Research Institute (IDRI), Seattle, United States. For the ELISA’s standardization, two positive (parasitologically positive) and one negative (parasitologically and DAT negative) samples were initially used. Table I shows the parameters tested using the different recombinant antigens. High binding polystyrene plates of 96 wells (half area) (Corning, Incorporated, New York, USA), were coated separately with each antigen (50 µL/well), diluted in 0.06M carbonate/bicarbonate buffer pH 9.6, being 0.5 µg/mL for rK39 and 1 µg/mL for rK28, rKR95 and rK18. After overnight at 4ºC, in a humid chamber, the plates were washed three times with 0.01M phosphate-buffered saline (PBS), pH 7.2 containing 0.05% Tween 20 (Polyoxyethylene-sorbitan monolaurate, Sigma-Aldrich, St. Louis, USA) (PBS-T). The plates were blocked with 125 µL/well of PBS-T containing 5% skimmed milk (Molico, Nestlé) (PBS-T-L 5%) at 37ºC for 2 h in a humid chamber, followed by three washes with PBS-T. For standardization, selected serum samples were diluted 1/25, 1/50 and 1/100 in PBS-T containing 2% and 5% skimmed milk (PBS-T-L 2% and 5%). After applying the sample dilutions in duplicate (50 µL/well), the plates were incubated at 37ºC in a humid chamber for 30 min, followed by five washes with PBS-T. The reaction was developed by incubating the plates with the TMB/H_2_O_2_ (Tetramethylbenzidine/hydrogen peroxide) chromogen (Novex-Life Technologies, Carlsbad, CA, USA) (50 µL/well) at four different times: 5, 7, 10 and 15 min. The reaction was stopped with 2N sulfuric acid (H_2_SO_4_) (25 µL/well). The absorbances were read at 450 nm using an ELISA reader (Multikan Go-Thermo Scientific, Finland).

**TABLE I:** Parameters tested for standardization of enzyme-linked immunosorbent assay (ELISA) with rK39, rK28, rKR95 and rK18

Tested parameters	
Polystyrene plate	Costar 3690 (96 wells, half area)
Antigen concentration	0.5 and 1.0 µg/mL
Sample and conjugate diluent	Skimmed milk at 2.0% and 5.0%
Sample dilution	1/25, 1/50, 1/100
Conjugate dilution	1/5,000, 1/10,000, 1/20,000, 1/30,000, 1/40,000, 1/50,000
Chromogen incubation time	5, 7, 10 and 15 min

- Evaluation of recombinant antigen-ELISA in laboratories within *Leishmania* endemic areas - Protocols were distributed to laboratories in *Leishmania* endemic regions and the technicians trained accordingly. ELISA with recombinant antigens were performed at the laboratory of the IMT/USP, the coordination center, and at laboratories in other locations (Laboratório de Leishmanioses da Faculdade de Medicina Veterinária da UFMT, IAL/SP, IAL/RP) and Laboratório de Infectologia Veterinária do Hospital-Escola de Medicina Veterinária da UFBA), where leishmaniasis caused by *L. infantum* is endemic. The results were compared to ensure the functionality of the tests, regardless of the location, provided they were carried out under appropriate conditions.

*Evaluation of samples from a follow-up study with dogs in the endemic area* - To evaluate if the studied assays can detect an early infection in animals, samples from 30 dogs from a cohort of 600 dogs from Bauru - SP collected at day 0 (time 1) and 180 days (time 2) of follow-up were evaluated. Samples from this cohort had been previously tested by the DPP + *L. major*-like-ELISA tests in Bauru - SP. The samples of the 30 dogs selected were submitted to rK39-ELISA, rK28-ELISA, and rKR95-ELISA. These dogs received collars impregnated with deltamethrin (Scalibor - Intervet) that were changed every 6 months or replaced in case of losses, to avoid infection through contact with the vector during the study.

*Evaluation of cross-reactivity with E. canis* - To evaluate if the recombinant antigens detect antibody related to other diseases, we selected 10 samples from dogs without clinical signs for CanL but reagent for *E. canis* in SNAP 4DX Plus^®^ test (IDEXX Laboratories Inc., Westbrook, ME, USA). These samples were non-reagent for *B. burgdorferi*, *A. phagocytophilum*, *A. platys* and *D. immitis*. The samples of the selected dogs were submitted to DPP, *L. major*-like-ELISA, DAT, rK39-ELISA, rK28-ELISA and rKR95-ELISA.

*Statistical analysis* - With the results of the absorbances obtained in each assay with specific recombinant antigens, the percentage of absorbance of the positive standard (ABS% Positive) was calculated by dividing the absorbance of each sample by the absorbance of the positive standard serum and multiplying by 100.^34^ The ROC curves were constructed using the ABS% Positive, obtaining the values of sensitivity, specificity and 95% confidence intervals and determining the cut-off. The agreement of the results of the techniques (two by two) was performed using the *Kappa* index. The stability of the sensitized plates was evaluated by linear regression of each sample. To compare the reactivity indices of samples with the recombinant antigens, the Friedman test (paired samples) was used, followed by Dunn’s multiple comparison test (nonparametric data). The comparison of the sensitivity and specificity of the methods used was made by McNemar’s chi-square test and the comparison between proportions by the Fisher’s exact probability test or the chi-square test. Statistical analysis was performed using the GraphPad Prism 5 (GraphPad Software Inc., San Diego, CA, USA) and SigmaStat 3.5 analysis system software (Systat Software, Richmond, CA, USA).

## RESULTS

*Initial sample characterization* - The samples were characterized according to the diagnostic algorithm recommended by the Brazilian Ministry of Health (MH). This involves initial evaluation with DPP followed by *L. major*-like-ELISA of DPP-reactive samples. When the sera of 74 symptomatic dogs with positive parasitological examination were submitted to the MH algorithm, the sensitivity was 93.2% (95% CI: 85.1-97.1%) and the specificity 100.0% (95% CI: 94.6-100.0%) with 66 samples from healthy dogs (all samples were negative in DPP).

**Fig. 3:**
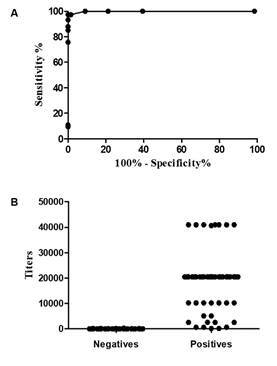
receiver operating characteristic (ROC) curve (A) and distribution of titers (B) of the direct agglutination test (DAT). ROC curve (A) constructed from the titers obtained in the DAT, assaying 74 samples from parasitologically positive dogs for *Leishmania infantum* infection and 66 from negative healthy dogs; (B) distribution of the titers of the samples tested.

We also characterized samples by their reactivity in DAT. The ROC curve was constructed from the titers of positive samples (dogs with *L. infantum* infection) and negative samples from healthy dogs, generating a sensitivity of 97.3% (95% CI: 90.6-99.7%) and specificity of 100.0% (95% CI: 94.6-100.0%) at a 1/640 cut-off point (Fig. 3).

Of the 74 parasitologically positive symptomatic VL dogs, the MH algorithm was positive in 69 (sensitivity 93.2%), while the DAT was positive in 72 (sensitivity 97.3%); one sample did not react in either test (McNemar’s test, p = 0.3711). Although the difference between the MH algorithm and DAT sensitivity was not significant, given the greater number of samples positive by DAT, we determined to use DAT as a reference to compare the ELISA performance using recombinant antigens.

*Performance of recombinant antigen ELISA* - In the standardization of each of the ELISA using recombinant antigens, the best conditions were established as: antigen concentration 0.5 µg/mL (rK39) and 1.0 µg/mL (rK28, rKR95 and rK18); serum dilution 1/25 (rK18) and 1/100 (rK39, rK28 and rKR95); conjugate dilution 1/40,000 (all antigens), chromogen time 5 min (rK39, rK28 and rKR95) and 7 min (rK18) (Table II).

**TABLE II:** Parameters chosen in the standardization of rK39-enzyme-linked immunosorbent assay (ELISA), rK28-ELISA, rKR95-ELISA and rK18-ELISA

Parameters	rK39	rK28	rKR95	rK18
Antigen concentration	0.5 µg/mL	1.0 µg/mL	1.0 µg/mL	1.0 µg/mL
Blocking solution volume	125 µL	125 µL	125 µL	125 µL
Concentration of skimmed milk in the blocking solution	5.0%	5.0%	5.0%	5.0%
Concentration of skimmed milk in serum diluent and conjugate	5.0%	5.0%	5.0%	5.0%
Sample dilution	1/100	1/100	1/100	1/25
Conjugate dilution	1/40,000	1/40,000	1/40,000	1/40,000
Volume of TMB/H_2_O_2_	50 µL	50 µL	50 µL	50 µL
TMB reaction time	5 min	5 min	5 min	7 min
Volume of H_2_SO_4_ (2N)	25 µL	25 µL	25 µL	25 µL

H2SO4: sulfuric acid; µg: microgram; mL: milliliter; µL: microliter; TMB/H_2_O_2_: Tetramethylbenzidine/hydrogen peroxide.

**Fig. 4:**
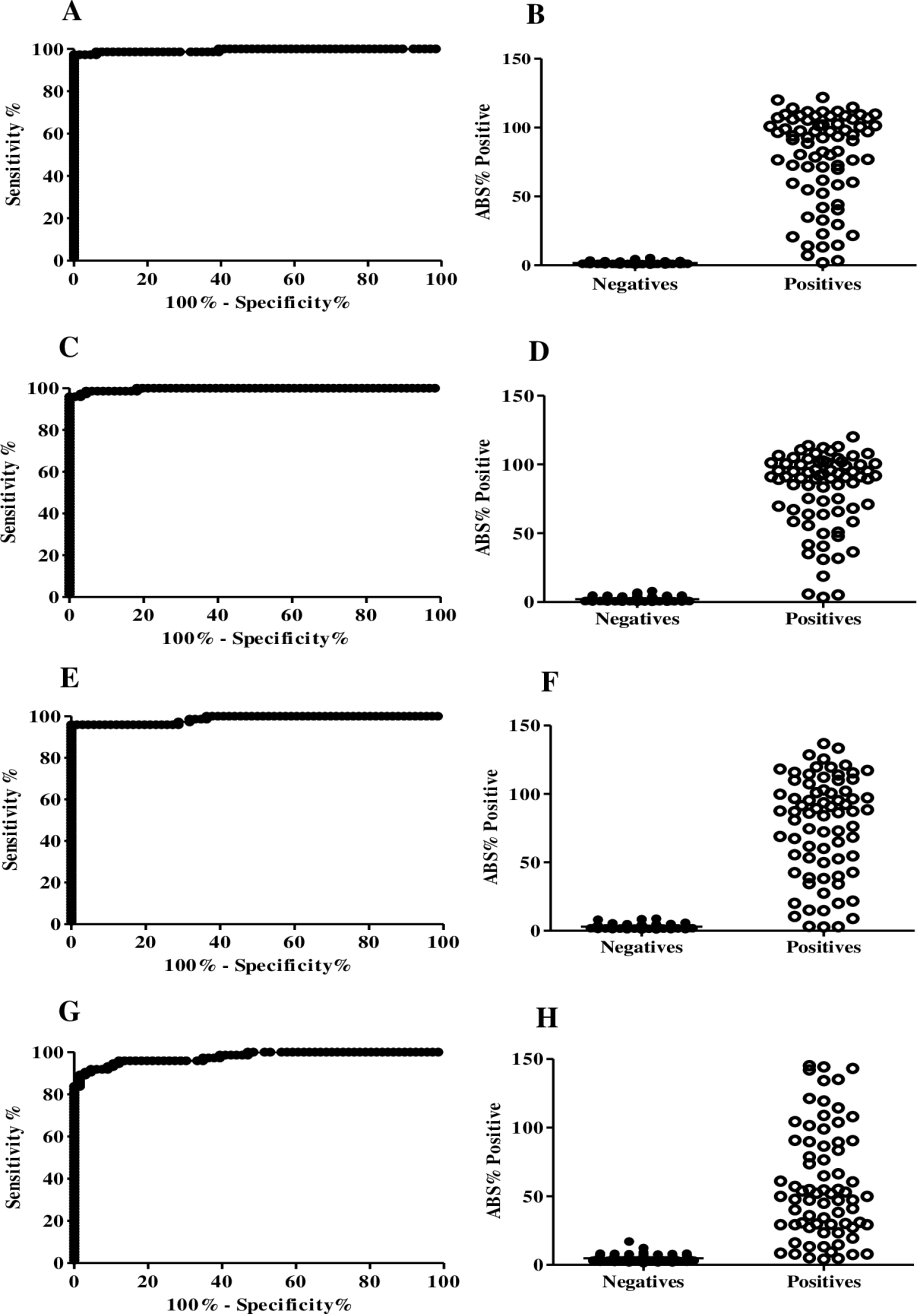
receiver operating characteristic (ROC) curves (A, C, E, G) and the percentage of absorbance of each sample in relation to the positive standard (ABS% Positive) values (B, D, F, H) of rK39-enzyme-linked immunosorbent assay (ELISA), rK28-ELISA, rKR95-ELISA, and rK18-ELISA. ROC curves were constructed from the values of ABS% Positive obtained in the rK39-ELISA (A, B), rK28-ELISA (C, D), rKR95-ELISA (E, F) and rK18-ELISA (G, H) for IgG antibodies, testing 74 samples of dogs parasitologically positive for *Leishmania infantum* and 66 of negative healthy dogs.

Based on the ROC curves constructed using the values of the ABS% Positive of the 74 samples of dogs with symptomatic VL and 66 samples of healthy dogs, the optimal cut-off point was 6.19 with rK39-ELISA; 13.42 with rK28-ELISA; 8.92 with rKR95-ELISA; 12.76 with rK18-ELISA. Using these cut-off values, the sensitivity (95% CI) of the ELISA using rK39, rK28, rKR95 and rK18 was, respectively, 97.3% (90.6-99.7%); 95.9% (88.6-99.2%); 95.9% (88.6-99.2%) and 89.1 (79.8-95.2%) and the specificity of 100.0% (94.6-100.0%); 100.0% (94.6-100.0%); 100.0% (94.6-100.0%) and 98.6% (91.8-100.0%) (Fig. 4).

Considering the 74 parasitologically positive symptomatic VL dogs, no significant difference was observed in the sensitivity of the MH algorithm in relation to the rK39-ELISA (McNemar’s test, p = 0.2482); both the rK28-ELISA and the rKR95-ELISA (McNemar’s test, p = 0.4795); and the rK18-ELISA (McNemar’s test, p = 0.5050).

*Sensitivity of recombinant antigen ELISA with subclinically VL infected dog samples -* As a selection criterion, the 19 samples of subclinically infected dogs were positive in the DAT and MH algorithm. In this group of asymptomatic dogs, the sensitivity (95% CI) of the rK39-ELISA, rK28-ELISA, rKR95-ELISA and rK18-ELISA was, respectively, 89.5% (68.6-97.1%); 84.2% (62.4-94.5%); 84.2% (62.4-94.5%) and 57.9% (36.3-76.9%). In this group of asymptomatic animals, we did not compare the sensitivity of the ELISA test with recombinant antigens and the MH algorithm because all animals in the panel had already been considered positive in the tests recommended by the Ministry of Health of Brazil.

Comparing the groups of symptomatic (74) and subclinically VL infected (19) dogs, no significant difference was observed in the sensitivity obtained with rK39-ELISA (Fisher’s exact test, p = 0.1841), rK28-ELISA (Fisher’s exact test, p = 0.0973) and rKR95-ELISA (Fisher’s exact test, p = 0.2694). However, lower sensitivity was observed in the subclinically VL infected group with rK18-ELISA (Fisher’s exact test, p = 0.0035).

*Evaluation of cross-reactivity with E. canis* - After verifying the good performance of the tests with the recombinant antigens, we evaluated the possibility of cross reaction with *E. canis*, a disease transmitted mainly by ticks and very common in the clinical practice of veterinary routine. Of the 10 animals evaluated, 2 (20.0%, 95% CI: 6.0-46.7%) dogs were reactive in the rKR95-ELISA, one (10.0%, 95% CI: 0.0-42.6%) in the DPP and non-reactive (0.0%, 95% CI: 0.0-32.1%) in *L. major*-like-ELISA, rK39-ELISA, rK28-ELISA and DAT.

**Fig. 5:**
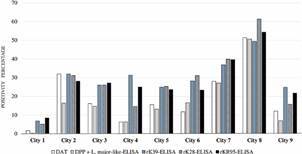
positivity (%) of the direct agglutination test (DAT), Brazilian Ministry of Health (MH) algorithm dual-path platform (DPP) + *Leishmania major*-like-enzyme-linked immunosorbent assay (ELISA), rK39-ELISA, rK28-ELISA, and rKR95-ELISA. Two thousand five hundred thirty samples from different regions of Brazil were assayed.

**Fig. 6:**
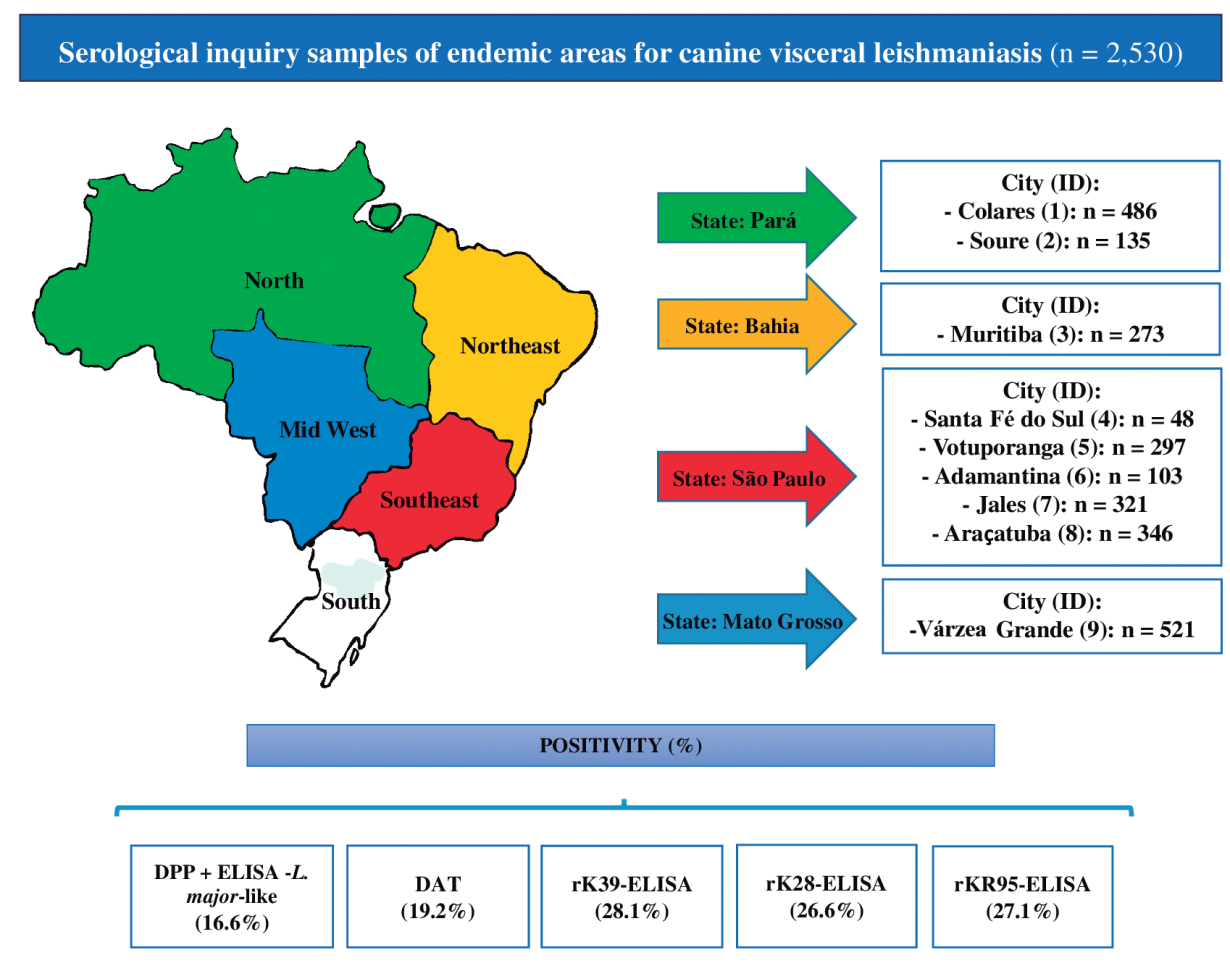
positivity of dual-path platform (DPP) + enzyme-linked immunosorbent assay (ELISA)-*Leishmania major*-like, direct agglutination test (DAT), rK39-ELISA, rK28-ELISA and rKR95-ELISA in 2,530 samples from serological inquiry done in various geographical regions. ID: city identification.

*Evaluation of each serological diagnostic strategy for the detection of L. infantum-infected dogs in various localities* - The percentages of the positivity of samples from each studied location, using the DAT, MH algorithm (DPP + *L. major*-like-ELISA), rK39-ELISA, rK28-ELISA and rKR95-ELISA are presented in Figs 5, 6, and Table III. DAT had significantly higher positivity than the MH algorithm in cities 1, 2, 9 (McNemar’s test, p = 0.0455, p = 0.0002, p < 0.0001, respectively), and total samples (McNemar’s test, p < 0.001). The positivity was higher in ELISA with recombinant antigens compared with DAT and MH algorithm in total samples (McNemar’s test, p < 0.001) and in most locations (McNemar’s test, p < 0.05). The exceptions were DAT versus rK39-ELISA, versus rK28-ELISA and versus rKR95-ELISA in city 2; DAT versus rK28-ELISA in city 4 and DAT versus rKR95-ELISA in city 8; MH algorithm versus rKR95-ELISA in cities 6 and 8; MH algorithm versus rK28-ELISA in city 4. Compared with rK39-ELISA, rK28-ELISA detected lower positivity in cities 4 and 9 (McNemar’s test, p = 0.0269, p < 0.0001, respectively). Compared with rKR95-ELISA, rK28-ELISA showed lower positivity in cities 1 and 9 (McNemar’s test, p = 0.0304, p = 0.0034, respectively). Compared with rK28-ELISA, rKR95-ELISA showed lower positivity in city 8 (McNemar’s test, p = 0.0014). There was no significant difference in positivity between rK39-ELISA and rKR95-ELISA (McNemar’s test, 0.0896 ≥ p ≤ 0.7548). All evaluations were performed with the recombinant antigen rK18, however, as its performance was worse than the other antigens of the study, its results were not presented.

**TABLE III:** Results of direct agglutination test (DAT), dual-path platform (DPP) + *Leishmania major*-like-enzyme-linked immunosorbent assay (ELISA), rK39-ELISA, rK28-ELISA and rKR95-ELISA of 2,530 serological inquiry samples

City	DAT		MH algorithm (DPP + *L. major* -like-ELISA)		rK39-ELISA		rK28-ELISA		rKR95-ELISA	
	R (%)	95%CI	R (%)	95%CI	R (%)	95%CI	R (%)	95%CI	R (%)	95%CI
City 1 (n: 486)	8 (1.6)	0.8-3.3	1 (0.2)	0.0-1.3	33 (6.8)	4.8-9.4	25 (5.1)	3.5-7.5	41 (8.4)	6.3-11.3
City 2 (n: 135)	43 (31.9)	24.6-40.1	22 (16.3)	10.9-23.5	43 (31.9)	24.6-40.1	42 (31.1)	23.9-39.4	38 (28.1)	21.2-36.3
City 3 (n: 273)	44 (16.1)	12.2-21.0	40 (14.7)	10.9-19.4	71 (26.0)	21.1-31-5	71 (26.0)	21.1-31.5	74 (27.1)	22.2-32.7
City 4 (n: 48)	3 (6.2)	1.5-17.5	3 (6.2)	1.5-17.5	15 (31.2)	19.9-45.4	7 (14.6)	6.9-27.5	12 (25.0)	14.8-38.9
City 5 (n: 297)	46 (15.5)	11.8-20.1	39 (13.1)	9.7-17.5	74 (24.9)	20.3-30.1	75 (25.3)	20.6-30.5	70 (23.6)	19.1-28.7
City 6 (n: 103)	12 (11.7)	6.6-17.9	17 (16.5)	10.5-24.9	29 (28.2)	20.3-37.5	32 (31.1)	22.9-40.6	24 (23.3)	16.1-32.4
City 7 (n: 321)	90 (28.0)	23.4-33.2	87 (27.1)	22.5-32.2	118 (36.8)	31.7-42.2	128 (39.9)	34.7-45.3	127 (39.6)	34.4-45.0
City 8 (n: 346)	178 (51.4)	46.2-56.7	175 (50.6)	45.3-55.8	200 (57.8)	52.5-62.9	212 (61.3)	56.0-66.3	188 (54.3)	49.1-59.5
City 9 (n: 521)	63 (12.1)	9.5-15.2	36 (6.9)	5.0-9.4	129 (24.8)	21.2-28.6	82 (15.7)	12.8-19.1	113 (21.7)	18.4-25.4
Total (n: 2530)	487 (19.2)	17.8-20.8	420 (16.6)	15.2-18.1	712 (28.1)	26.4-29.9	674 (26.6)	24.9-28.4	687 (27.1)	25.5-28.9

MH: Brazilian Ministry of Health; R: reactive; 95% CI: 95% confidence interval; n: number of samples.

*Evaluation of the prognostic ability of recombinant antigen ELISA* - Since ELISA using recombinant antigens showed higher sensitivity than both the DAT and the MH algorithm, we considered the possibility that the ELISA would allow an earlier detection of the infection than the currently recommended assays. To test this, we evaluated samples’ reactivity from a cohort of 600 dogs sampled over an extended period. Considering the MH algorithm results, samples from 30 dogs were selected and distributed in two groups with 15 dogs in each. Group 1 consisted of 15 dogs that at time 1 were considered negative at the initial evaluation for *Leishmania* infection by the MH algorithm but subsequently became positive six months later (time 2). Group 2 consisted of samples from 15 dogs that seroconverted from DPP negative at time 1 to DPP positive at time 2 but were negative by *L. major*-like-ELISA at both periods.

**Fig. 7:**
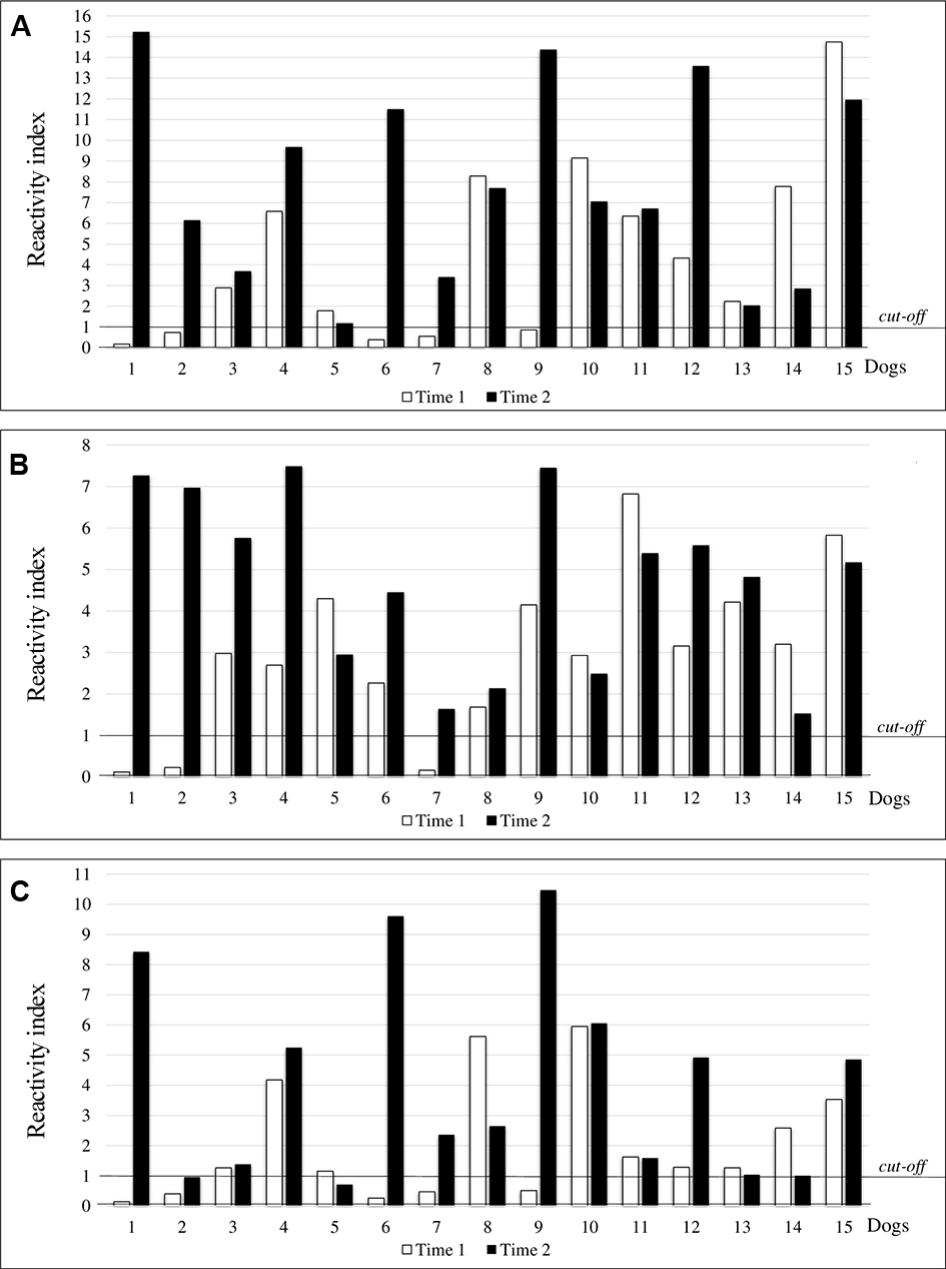
reactivity indices of samples from 15 dogs in collections at days 1 (time 1) and 180 (time 2) using the rK39-enzyme-linked immunosorbent assay (ELISA) (A), rK28-ELISA (B) and rKR95-ELISA (C). Group 1 - dogs considered negative for *Leishmania* infection by Brazilian Ministry of Health (MH) algorithm at time 1 that became positive at time 2.

In Group 1, the rate of positive responses at time 1 were determined to be 66.7% by rK39-ELISA; 80.0% by rK28-ELISA and 60.0% by rKR95-ELISA (chi-square test, p = 0.4839) (Fig. 7). Differences in reactivity signal intensity were observed at time 1 using different recombinant antigens (Friedman test, p = 0.0045; Dunn’s multiple comparison test, p < 0.05), with a significantly higher median reactivity index observed in the rK39-ELISA (2.89) and rK28-ELISA (2.97) than rKR95-ELISA (1.26). At time 2, all dogs were seropositive by MH algorithm, rK39-ELISA and rK28-ELISA; 80.0% by rKR95-ELISA. There was also a great difference in the reactivity indices among the recombinant antigens (Friedman test, p = 0.0010; Dunn’s multiple comparison test, p < 0.05), with a significantly higher median reactivity index obtained with rK39-ELISA (7.03) than both rK28-ELISA (5.16) and rKR95-ELISA (2.64).

**Fig. 8:**
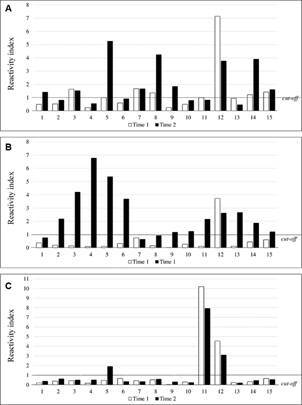
reactivity indices of samples from 15 dogs in collections at days 1 (time 1) and 180 (time 2) using the rK39-enzyme-linked immunosorbent assay (ELISA) (A), rK28-ELISA (B) and rKR95-ELISA (C). Group 2 - dogs considered negative for *Leishmania* infection by the Brazilian Ministry of Health (MH) algorithm at time 1 and that became positive only by dual-path platform (DPP) at time 2.

In group 2, rK39-ELISA assessed that 47.0% of the dogs were seropositive at time 1. Only one sample was positive by rK28-ELISA and two by rKR95-ELISA (chi-square test, p = 0.0057) (Fig. 8). At time 2, 60.0% of the dogs were positive by rK39-ELISA, 80.0% by rK28-ELISA and 20.0% by rKR95-ELISA (chi-square test, p = 0.0036). Thus, the lowest median reactivity was observed in rKR95-ELISA (0.50), significantly lower than that of both rK39-ELISA (1.52) and rK28-ELISA (2.16) (Friedman test, p = 0.0007; Dunn’s multiple comparison test, p < 0.05).

*Viability to use rK39-ELISA, rK28-ELISA and rKR95-ELISA in the laboratories of the endemic area* - Having demonstrated the utility of these ELISA in our laboratory, we then assessed assay robustness by distributing them to laboratories in areas endemic for CanL after specific training of technicians. The agreement between the UFMT and the IMT was perfect (κ = 1.00, 95% CI = 1.00-1.00) in the rK39-ELISA, rK28-ELISA and rKR95-ELISA tests and very good (κ = 0.92, 95% CI = 0.81-1.00) in the rK18-ELISA. The agreement between the IMT and the IAL/SP was perfect (κ = 1.00, 95% CI = 1.00-1.00) in the rK39-ELISA, rK28-ELISA, rKR95-ELISA, and rK18-ELISA. The agreement between the IMT and the IAL/RP was perfect (κ = 1.00, 95% CI = 1.00-1.00) in the rK39-ELISA, rK28-ELISA and rKR95-ELISA and very good (κ = 0.86, 95% CI = 0.71-1.00) in the rK18-ELISA. The agreement between the IMT and the UFBA was good in the rK39-ELISA (κ = 0.62, 95% CI = 0.36-0.88), rK28-ELISA (κ = 0.62, 95% CI = 0.39-0.86) and rK18-ELISA (κ = 0.64, 95% CI = 0.38-0.90).

**Fig. 9:**
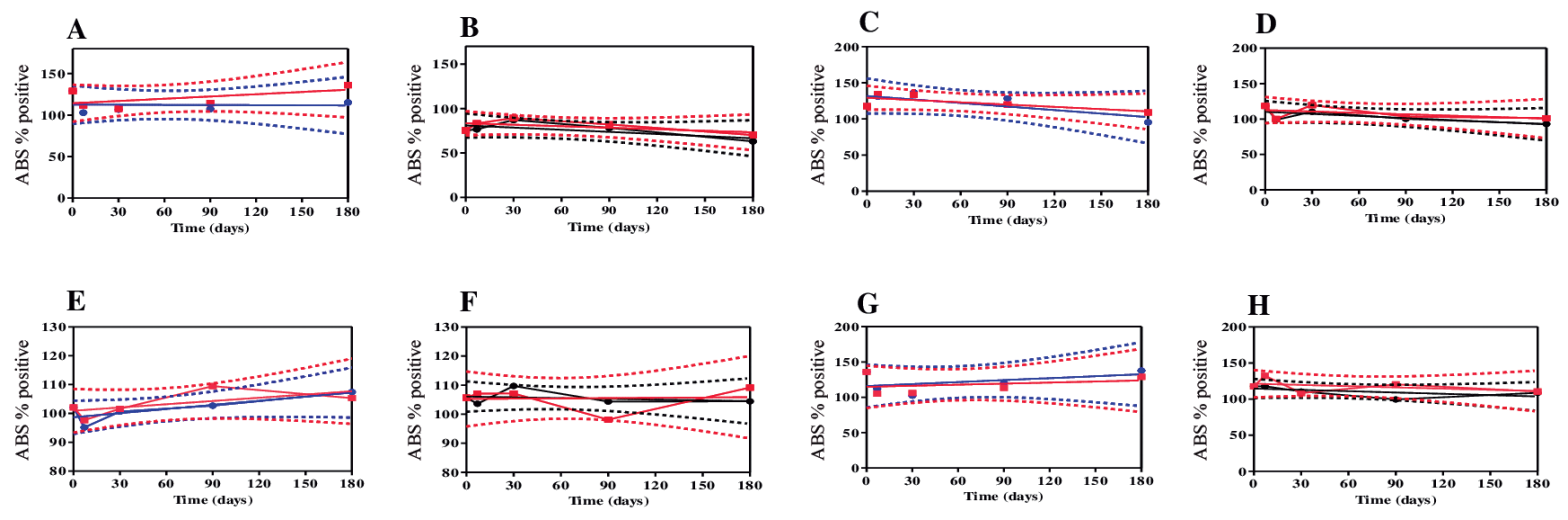
linear regression to study the stability of pre-sensitized plates in the rK39-enzyme-linked immunosorbent assay (ELISA) (A, B), rK28-ELISA (C, D), rKR95-ELISA (E, F) and rK18- ELISA (G, H), at 4°C and -20°C, respectively. Linear regression graphs between the percentage values of absorbance of each sample in relation to the positive standard and the storage time of the plates at temperatures of 4°C and -20°C obtained in the rK39-ELISA (A, B), rK28-ELISA (C, D), rKR95-ELISA (E, F) and rK18-ELISA (G, H) tests in comparison to the control. The tests were performed on days 0, 7, 30, 90 and 180. For each antigen test, a duplicate negative control sample, a duplicate positive control sample and a panel with 20 duplicate positive samples were used. Dotted lines indicate the 95% confidence interval (95% CI). ABS % Positive: the percentage of absorbance of each sample relative to the positive standard.

Meanwhile, we ascertained that the plates pre-sensitized with recombinant antigens and stored at 4ºC and -20ºC were stable with no significant decrease in reactivity up to 180 days (Fig. 9).

## DISCUSSION

Considering the fundamental role of dogs in the VL epidemiology, diagnostic algorithms are proposed for routine surveillance to detect *L. infantum*-infected dogs in control programs. Previous studies show unsatisfactory performance^9^ ^,^ ^35^ of the currently used assays in the diagnostic routine in Brazil. This study evaluated ELISA using four different recombinant antigens, rK39, rK28, rKR95 and rK18, hypothesizing that these could provide better diagnostic performance relative to the current strategy. We obtained excellent results with the rK39 antigen (97.3% sensitivity and 100.0% specificity), while rK18-ELISA performed poorly in the analysis. Results generated with recombinant proteins rK28 and KR95 were also satisfactory, with sensitivity and specificity values only slightly lower than those observed with rK39. The results of sensitivity and specificity obtained in our study with the rK39 antigen are close to those found by Scalone et al.^21^ (sensitivity of 97.1% and specificity of 99.4%) who evaluated Italian dogs in ELISA test. Different from these results, in a study carried out by Pereira et al.,^36^ the rK39-ELISA test showed a lower sensitivity of 82%. In that evaluation, the selection criterion of infected animals was only by serological methods (DPP + *L. major*-like-ELISA), not being used another method for the confirmation of the infection, leaving a margin of doubt about the real infection of these dogs that could thus interfere in the sensitivity results. Importantly, when samples from a group of early-infected still subclinical dogs were evaluated, the accuracy (95% CI) of ELISA with recombinant K39, K28, KR95 and K18 was 97.7% (91.6-99.9%), 96.6% (90.0-99.2%), 97.7% (91.6-99.9%) and 84.1% (74.9-90.4%), respectively. These data suggest the detection of infected dogs before the emergence of overt clinical signs.

As the main purpose of the test is its use in serological inquiry in endemic areas for VL, we conducted a search with 2,530 canine samples collected for serological inquiries in four Brazilian states. Although the parasitological examination is the gold standard for CanL diagnosis, in serological surveys of endemic areas, it becomes impracticable to perform this method due to the demand of execution time, invasive collection material and specialized team. Thus, in our study, we used the DAT, which shows good diagnostic performance in CanL, as a reference test for comparing survey samples. We submitted the samples to the MH algorithm and additionally to the DAT. The reactivities were 28.1% for rK39-ELISA, 27.1% for rKR95-ELISA, and 26.6% for rK28-ELISA, compared with 16.6% with the MH algorithm. These data suggested that the tests with these recombinant antigens detect earlier the dogs with subclinical infections. We evaluated a situation substituting total *L. major*-like ELISA by rK39-ELISA in the MH algorithm, then the reactivity reached 17.4%, thus similar, tending to be higher in this comparison. In both evaluations, both in DPP + *L. major*-like-ELISA and DPP + rK39-ELISA, the concordances with the DAT, the reference test, were good. Since the total antigen production is a limiting factor in producing diagnostic kits for national demand, the use of recombinant antigens for ELISA kits emerges as a real possibility to replace *L. major*-like-ELISA.

Analyzing the performance of the different tests in samples obtained from different geographical areas, endemic for zoonotic VL, we observed that the presently used assays had non-satisfactory performance, compared with tests based on recombinant antigens (K39, K28 and KR95), in city 1 (PA), city 2 (PA), city 3 (BA), city 5 (SP), city 7 (SP) and city 9 (MT). The reason may be the intra-specific variability of *L. infantum* in these locations. The ELISA with recombinant antigens K39, K28 and KR95 showed good and similar performance in the global analysis. Still, analyzing the samples according to geographical regions, we observed variable performances with rK28-ELISA and rKR95-ELISA being worse than rK39-ELISA that showed good performance regardless of the geographic area. Thus, rK39-ELISA appears to be a good assay for serological inquiry in any region. We should consider further that the recombinant antigen K39 is based on the sequence of *L. infantum* (syn. *L. chagasi*),^15^ a species causing zoonotic VL in Brazil. To reinforce our view that recombinant antigens are highly specific, data by Scalone et al.^21^ show no cross-reactivity with other diseases using rK39-ELISA. In the study carried out by Zanette et al.,^37^ there was cross-reactivity with *E. canis*, *Toxoplasma gondii* and *Neospora caninum* using the Kalazar Detect™ test, which uses the rK39 antigen. However, in our study, the 10 sera of dogs positive for *E. canis* did not react in the ELISA with the rK39 and rK28 antigens. Although the number of samples was small, these results may discard the possibility of cross-reactivity at least with *E. canis* of samples positive for rK39-ELISA and rK28-ELISA.

With rKR95-ELISA, although the protein identity of rKR95 was 79% with *T. cruzi*, no cross-reactivity was seen with human patients’ sera.^26^ In our study, there was a cross-reaction with two of the ten dogs positive for *E. canis*.

On the data showing higher positivity of ELISAs with recombinant antigens, around 10% higher than the result obtained with the MH algorithm, we considered indicative of the existence of a considerable number of false-negative results.

To address this possibility, we evaluated animals from a cohort of 600 dogs from an endemic area for *L. infantum* infection submitted to periodic evaluation. The dogs in this part of the study were previously evaluated through physical examination and serology (MH algorithm). From the negative diagnosis (DPP + *L. major*-like-ELISA), the animals received collars impregnated with deltamethrin (Scalibor- Intervet) that were changed according to the manufacturer’s recommendation or replaced in case of losses to avoid infection through contact with the vector. Thus, the risk of infection by the vector was extremely reduced. Despite being the gold standard in the diagnosis of CanL, the parasitological examination presents variable sensitivity,^38^ especially in asymptomatic animals,^39^ so we do not evaluate these animals parasitologically.

We selected 15 dogs (Group 1) that were negative using the DPP test at time 1 that became positive using the MH algorithm six months later (time 2). Although the number of samples was reduced, the results were conclusive. Using rK39-ELISA, around 67% of the dogs turned positive among those considered negative by the MH algorithm at time 1. We also evaluated another 15 dogs from the same cohort that were initially negative in DPP in time 1 that turned out to become positive in DPP at time 2 (Group 2). Using rK39-ELISA, around 47% of these samples were positive, bringing concern on the performance of immunochromatographic rapid assays that are in use worldwide, although it was used here in one brand of kit.

These results suggest that ELISAs with *Leishmania* recombinant antigens detect infections earlier than the MH algorithm in use. All animals in group 1 in the second collection were reactive to the tests recommended by the Ministry of Health and we believe that some were already infected since the first collection, since anti-*Leishmania* antibodies were detected with the antigens used in our study. We may rule out the possibility of false positive results using ELISA with recombinant antigens considering the results of the present study showing above the analysis with positive samples confirmed by parasitological exams, and the high specificity obtained. These data bring concerns on the efficacy of measures to control *L. infantum* transmission, implying the maintenance of infected dogs in the disease cycle with a consequent risk for the human and canine population.

To ensure the ELISAs functionality in other laboratories, we evaluated the viability of applying the tests in laboratories of endemic areas for CanL. For this, we counted on the partnership of collaborating institutions that carried out the tests. The results of the concordance analysis were good to perfect and thus, we conclude that the standardized tests at the coordination center IMT can be safely reproduced by other laboratories provided they are submitted to appropriate test conditions. Further, the antigen-coated plates were stable up to 180 days when maintained at -20ºC and 4ºC.

In Brazil, the Leish-Tec^®^ vaccine is the only one approved for the prevention of Canine Visceral Leishmaniasis, and several debates regarding serological methods for the differentiation of vaccinated and infected dogs have been raised. In our study we did not evaluate groups of vaccinated dogs, but we believe that the antigens validated in the present study do not detect antibodies from animals vaccinated in the ELISA because they have different epitopes. In the study conducted by Campos et al.,^40^ dogs vaccinated with Leish-Tec^®^ were not reagent to DPP and *L. major*-like-ELISA in the 30, 180 and 360 days after the vaccination protocol.

In this study, we validated the ELISA with recombinant *Leishmania* antigens, suggesting the rK39 antigen for the diagnosis of CanL. This test is efficient, and it seems there is a superiority in sensitivity compared with the tests currently in use in Brazil. Thus, rK39-ELISA used as a single screening and the confirmatory test is a concrete possibility for the diagnosis of dogs infected with *L. infantum*, both in Brazil and in other locations where the dog is the reservoir. Although the control of CanL in Brazil was our primary interest, considering the increasing importance of VL caused by *L. infantum* in other parts of the world, the rK39-ELISA here shown with high diagnostic performance can be extended to the diagnosis and management of CanL worldwide.
